# Synthesis and Fungicidal Activity of β-Carboline Alkaloids and Their Derivatives

**DOI:** 10.3390/molecules200813941

**Published:** 2015-07-31

**Authors:** Zhibin Li, Shaohua Chen, Shaowen Zhu, Jianjun Luo, Yaomou Zhang, Qunfang Weng

**Affiliations:** 1 Key Laboratory of Natural Pesticide and Chemical Biology, Ministry of Education College of Agriculture, South China Agricultural University, Guangzhou 510642, China; E-Mails: zhibun_li@163.com (Z.L.); csh-happy@163.com (S.C.); chuk_shiuman@163.com (S.Z.); luojianjun@scau.edu.cn (J.L.); 2 College of Materials and Energy, South China Agricultural University, Guangzhou 510642, China; E-Mail: zhangyaom@163.com

**Keywords:** β*-*carboline, fungicidal activity, structure–activity relationship

## Abstract

A series of β-Carboline derivatives were designed, synthesized, and evaluated for their fungicidal activities in this study. Several derivatives electively exhibited fungicidal activities against some fungi. Especially, compound **F5** exhibited higher fungicidal activity against *Rhizoctonia solani*(53.35%) than commercial antiviral agent validamycin (36.4%); compound **F16** exhibited high fungicidal activity against *Oospora citriaurantii* ex Persoon(43.28%). Some of the alkaloids and their derivatives (compounds **F4** and **F25**) exhibited broad-spectrum fungicidal activity. Specifically, compound **F4** exhibited excellent high broad-spectrum fungicidal activity *in vitro*, and the curative and protection activities against *P. litchi in vivo* reached 92.59% and 59.26%, respectively. The new derivative, **F4**, with optimized physicochemical properties, obviously exhibited higher activities both *in vitro* and *in vivo*; therefore, **F4** may be used as a new lead structure for the development of fungicidal drugs.

## 1. Introduction

Plant pathogenic microorganisms can infect crops, causing local or whole plant disease and leading to significant economic losses. How to control them in modern agriculture is still a big challenge. Many kinds of fungicides are used to prevent and cure the diseases caused by fungi; however, these chemical agents cannot fully protect the crops or completely cure the crops’ tissues from fungal infection under field conditions. Therefore, novel and more practical fungicidal reagents are urgently needed.

**Figure 1:**
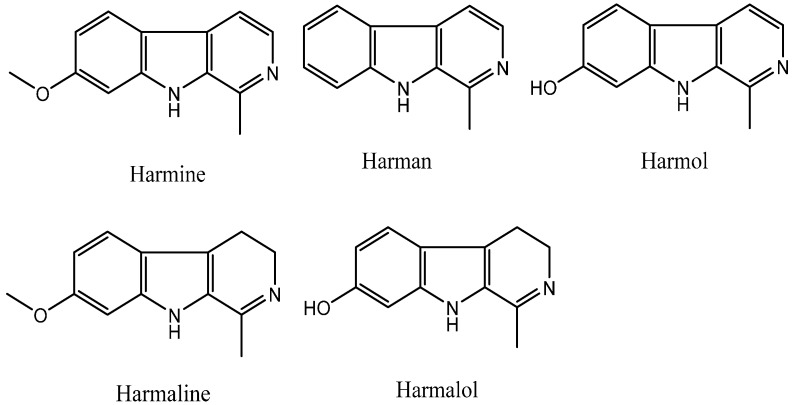
Chemical structures of β-carboline alkaloids.

Plants can produce some secondary metabolites with insecticidal, antifungal, or antibacterial biological activity; therefore, natural products can be used as ideal lead structures to develop agrochemicals. The β-carboline alkaloids are a large group of natural and synthetic indole alkaloids that possess a common tricyclic pyrido [3,4-*b*] indole ring structure (Figure 1) [1,2,3]. Harmine, harman, harmol, harmaline, and harmalol, which are β-carboline and dihydro-β-carboline alkaloids, are four representative harmala alkaloids. Harmine was originally isolated from *Peganum harmala* L*.* [4], and found to exhibit a cytotoxic effect on HL60 and K562 leukemic cell lines [5]. Harmane has DNA intercalation ability, leading to not only intercalation into DNA [6,7] and formation DNA adducts [8], but also inhibition of Topo I [7,9], Topo II [9], and MAO-A activity [10,11]. Harmol can induce autophagy and suppression of survivin expression, subsequently induce apoptotic cell death in U251MG human glioma cells [12] and apoptosis by caspase-8 activation independently from Fas/Fas ligand interaction in human non-small cell lung cancer (NSCLC) H596 cells [13], and significantly inhibit the dioxin-mediated induction of CYP1A1 at mRNA, protein, and activity levels in a concentration-dependent manner in human and murine hepatoma cells [14]. Harmaline can inhibit DNA excision repair [15], human DNA Topo I activity [7], PKC activity [16], and TMV [17] and against the amastigote stage of *Leishmania* [16]. Harmalol is able to induce melanogenesis through p38 MAPK signaling [18] and can act as an agent for preventing dioxin-mediated effects [19].

β-Carboline and its structural analogues in the medical and pharmaceutical are a research focus. However, there is limited information about these chemicals in agricultural areas and they lack system development and application. The β-carboline amides, containing amides and a carboline structure, represent a new direction for the development of plant-derived bio-pesticides. In addition, the antifungal activity of β-carboline will change when the 3-position of β-carboline was substituted [17,20]. In this study, their fungicidal activities were systematically evaluated. To investigate the biological activities of the substituents, β-carbolines containing different substituents were synthesized and their fungicidal activities were also systematically evaluated.

## 2. Results and Discussion

### 2.1. Synthesis

**Figure 2:**
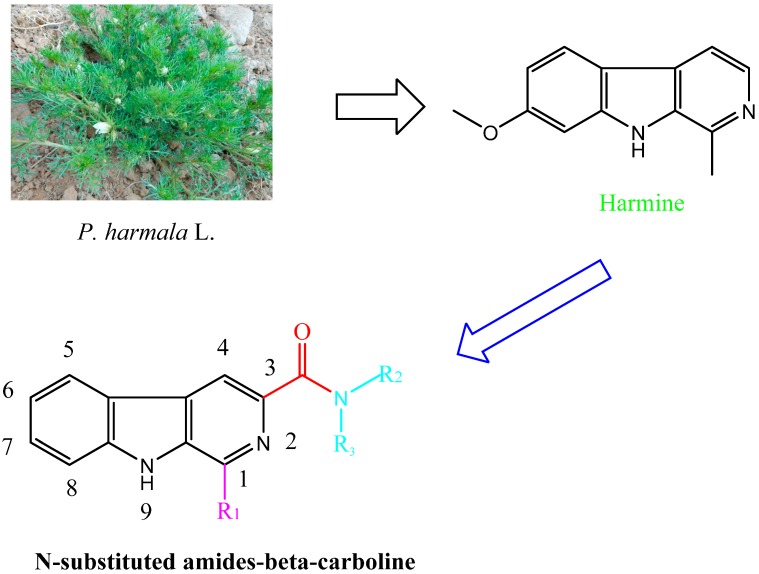
Design of target compounds.

**Scheme 1:**
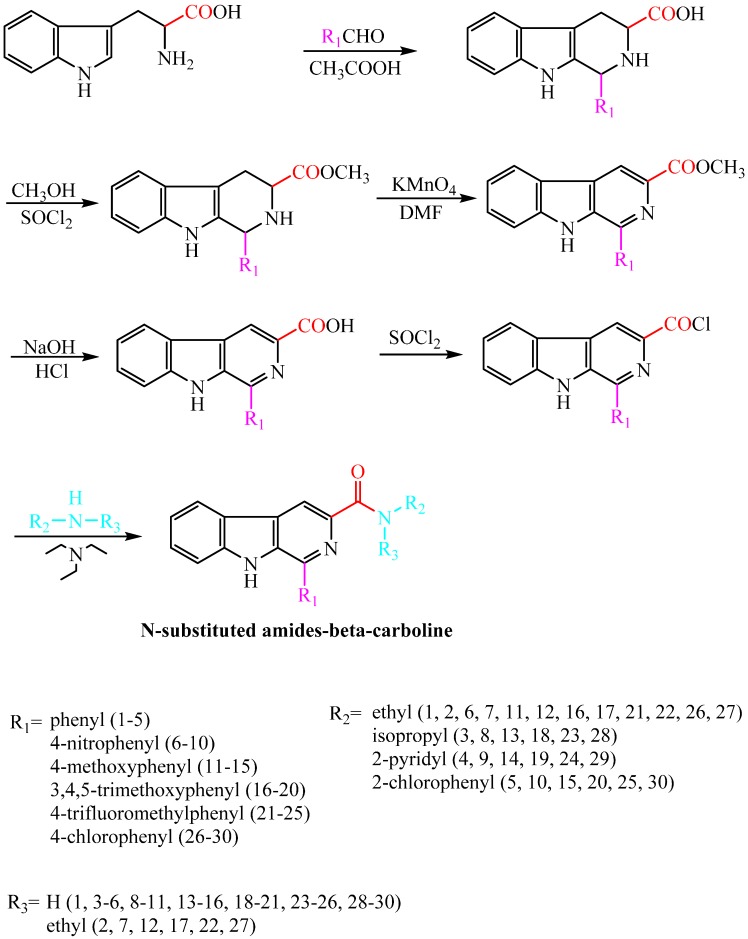
Synthesis of target compounds.

Compounds harmine, harmane, harmaline, and harmalol were obtained from Sigma-Aldrich, St. Louis, MI, USA. Their chemical structures are shown in Figure 1. Previous structure–activity relationship studies had demonstrated the influence of substituents in positions-1, -3, and -9 of the β-carboline skeleton for a variety of synthetic β-carboline derivatives [21,22,23,24]. In order to study the effect of main structure and the substituent groups at position 1 and 3 on their herbicidal activity, we synthesized a series of 30 novel β-carboline derivatives bearing a substituted amide group at C-3 and substituted groups at C-1 (Figure 2 and Scheme 1). All these compounds were characterized by their melting point, mass, infrared, IR, and ^1^H-NMR spectra, which confirmed the proposed structures of the new compounds. Tryptophan, which has an electron-rich indole ring, was used as the parent material when applying Pictet–Spengler or Bischler–Napieralski reactions [25] with a variety of aromatic aldehyde cyclization to give tetrahydrocarboline compounds, then oxidizing to obtain β-carboline compounds by using DMF as a solvent and KMnO_4_ as an oxidant. Pictet–Spengler reactions that used acetic acid as the catalyzed solvent produced a reaction that was refluxed at 80 °C to obtain a higher yield (above 80%) of tetrahydro-β-carboline compounds. Since the reaction temperature was moderate and the by-product generated was less, the product could be obtained with a purity of more than 90% by suction filtration and washing. Then the product could be used directly in the next reaction after drying. The carboxyl on the 3-position of tetrahydro-β-carboline must be protected by esterification, due to the fact that a carboxyl with high reaction activity could be easily decarboxylated, thereby losing carbonyl in the potassium permanganate conditions. Using KMnO_4_ to oxidize tetrahydro-β-carboline derivatives produced a lower yield, but the reagent was relatively inexpensive, and the reaction was easy to operate in the laboratory. The acylation reaction of amide synthesis used acid halide with ammonia or amine. Step 1: β-carboline-3-carboxylic acid reacted with an excess of thionyl chloride to become the corresponding acid chloride in the situation of catalyzer MDF of 1‰. The excess of thionyl chloride was both reactant and reaction solvent in the reaction, which may also remove water to reduce moisture in the system requirements of dry operation. HCl gas and SO_2_ gas were generated by the reaction; we utilized lye to absorb them. Step 2: acid chloride reacted with the corresponding amine. The reaction requires adding week base to neutralize the HCl so as to avoid amine reacting with HCl to generate the amine hydrochloride, which does not participate in the reaction. Because the reaction is intense, the product of the first step should be dissolved firstly in methylene chloride, and added slowly dropwise to the mixture of amine and triethylamine that was placed on an ice bath, and then the mixture stirred at room temperature for half an hour.

### 2.2. Fungicidal Activities

*Fungicide Screening*. Compounds **F1–30** were evaluated in a series of *in vitro* fungicidal tests, against a range of phytopathogenic species. The resulting data (Table 1) revealed that these alkaloids and their derivatives displayed potential fungicidal activity against six kinds of plant fungi including *F. oxysporum* f.sp.cubense, *C. gloeosporioides* (Penz.), *R. solani*, *P. litchii*, *P. nicotianae*, and *O. citriaurantii* ex Persoon. When the R_1_ was phenyl, the compounds exhibited higher activities than those with 4-nitrophenyl, 4-methoxyphenyl,3,4,5-trimethoxyphenyl,4-trifluoromethylphenyl, or 4-chlorophenyl as the R_1_. When the skeleton was β-carboline, the types of the substituents on the R_2_ and R_3_ had a significant influence on the fungicidal activities. For instance, compound **F4** (*N*-(2-pyridyl)-1-phenyl-9*H*-pyrido[3,4-*b*]indole-3-formamide) exhibited excellent fungicidal activity against most of the tested fungi, whereas compound **F2** (*N*,*N*-diethyl-1-phenyl-9*H*-pyrido[3,4-*b*]indole-3-formamide) exhibited only moderate activity; compound **F5**, containing a 4-trifluoromethylphenyl group, exhibited higher fungicidal activity than the compound **F1** and the group containing 4-chlorophenyl. Several of these compounds selectively exhibited fungicidal activities against some fungi. For example, the activities of harmine (against *F. oxysporum* f.sp.cubense and *R. solani*), compound **F16** (against *O. citriaurantii* ex Persoon), and compound **F25** (against *C. gloeosporioides* (Penz.)) were much higher than that against other fungi and were higher than the other compounds against some fungi, as shown in Table 1.

**Table 1:** Fungicidal activities of harmine, harmane, harmaline, harmalol, and compounds **F1**–**30** against six kinds of fungi (percent inhibition, %; 100 mg/L).

Compounds	*FO*	*CG*	*RS*	*PL*	*PN*	*OC*
Harmaline	32.9	19.7	38.4	49.4	30.2	10.7
Harmalol	12.3	21	25	30.4	20.4	5.8
Harmine	41.6	27.3	71.9	78.8	9.3	10.4
Harmol	33.3	13.8	27.8	46.7	38.4	10.2
**F1**	23	31.5	52.9	27.4	19.5	13.5
**F2**	0.2	4.6	9.9	- *^d^*	-	0.3
**F3**	-	3.2	14.9	-	18.6	-
**F4**	30.2	32	43.2	95.9	63.3	8.5
**F5**	19.9	30.5	53.4	76.3	31.7	6.8
**F6**	0.5	1	15.7	32.1	19	-
**F7**	3.1	10.2	15.7	2.1	9.8	0.5
**F8**	-	16.5	23.8	32.4	23.6	-
**F9**	-	19.6	15.1	53	36.4	-
**F10**	-	2.8	9.1	57.7	42.4	3.4
**F11**	2.8	20.1	9.4	35.6	23.4	6.8
**F12**	3.4	11.4	7.3	8.2	9.6	-
**F13**	9.9	11	36.6	/ *^e^*	/	/
**F14**	0.5	14	18.6	50.1	11.1	2.9
**F16**	7.2	7.9	31.9	3.7	-	43.3
**F17**	13.1	12.9	18.1	62.3	46.3	17.9
**F18**	10.2	16.4	38.7	23	2.8	6.6
**F19**	-	5.1	2.2	17.5	-	2.4
**F20**	1.2	12.3	7	68.4	55.4	15.3
**F21**	2.5	13.6	0.3	6.9	-	4.3
**F22**	6.1	7.8	2	40.4	-	3.3
**F23**	0.5	12.4	3.4	25.9	-	2.1
**F24**	20.8	39.6	14.5	76.6	53.7	8.7
**F25**	24.6	43.1	26	82.5	52.1	0.3
**F26**	3.4	15.1	11.8	20. 3	13.9	-
**F27**	17.1	28.4	30.7	75.8	54.4	8.5
**F28**	10.3	14.4	22.1	37.1	14.4	-
**F29**	6.1	34.8	26.4	/	/	/
**F30**	-	16.7	34	69.8	44	2.6
*^a^* Metalaxyl	/	/	/	93.4	84.1	/
*^b^* Validamycin	/	/	36.4	/	/	/
*^c^* Carbendazim	89	100	/	/	/	88.1

*^a^* Metalaxyl at 10 mg/L; *^b^* Validamycin at 100 mg/L; *^c^* Carbendazim at 50 mg/L; *^d^* “-”means no activity; *^e^* “/”means not tested. *FO* means *F. oxysporum* f.sp.cubense; *CG* means *C. gloeosporioides* (Penz.); *RS* means *R. solani*; *PL* means *P. litchi*; *PN* means *P. nicotianae*; *OC* means *O. citriaurantii* ex Persoon.

### 2.3. Leaf-Piece Assays

The alkaloids and their derivatives compounds **F1**–**30** were evaluated in *in vivo* fungicidal tests (litchi leaf-piece assays), against the phytopathogenic of *P. litchii*. The resulting data (Table 2) revealed that several compounds displayed potential fungicidal activity. When the R_1_ was4-nitrophenyl, these compounds exhibited higher preventative or curative activities than those with phenyl, 4-methoxyphenyl, 3,4,5-trimethoxyphenyl, 4-trifluoromethylphenyl, or 4-chlorophenyl as the R_1_. For example, compounds **F9** and **F10** showed higher preventative activity against *P. litchi* on litchi leaf-piece assays; compound **F6** had better curative activity against *P. litchi* on litchi leaf-piece assays. The types of the substituents on the R_2_ and R_3_ had a significant influence on the fungicidal activities. For instance, the ethyl group (**F1**, **F6**, **F11**, **F16**, **F21**, and **F26**) and the isopropyl group (**F3**, **F8**, **F13**, **F18**, **F23**, and **F28**) displayed higher curative activity than preventative, and compound **F11** had the highest curative activity against *P. litchi* on litchi leaf-piece assay. Comparatively, the *2-pyridyl* group (**F4**, **F9**, **F14**, **F19**, **F24**, and **F29**) and the *2-chlorophenyl* group (**F5**, **F10**, **F15**, **F20**, **F25**, and **F30**) exhibited higher preventative activity, and compound **F4** not only showed excellent preventative activity (92.6%) much better than the activity of metalaxyl (59.3%) against *P. litchi* on litchi leaf-piece assay, but also had a good curative activity (59.3%) against *P. litchi*. All the alkaloids and their derivatives had no phytotoxicity to the leaf pieces on the assay.

**Table 2:** Fungicidal activities of compounds **F1**–**30** against *P. litchi* by litchi leaf-piece assays (percent inhibition, %; 100 mg/L).

Compound	Preventative	Curative
Harmaline	0	44.4
Harmalol	0	7.4
Harmine	22.2	7.4
Harmol	7.4	51.6
**F1**	7.4	37
**F2**	14.8	29.6
**F3**	14.8	29.6
**F4**	92.6	59.3
**F5**	44.4	44.4
**F6**	0	51.9
**F7**	29.6	37
**F8**	7.4	37
**F9**	66.7	29.6
**F10**	51.9	0
**F11**	22.2	63
**F12**	22.2	33.3
**F13**	/ *^b^*	/
**F14**	0	22.2
**F16**	14.8	44.4
**F17**	37	0
**F18**	7.4	51.9
**F19**	0	44.4
**F20**	22.2	22.2
**F21**	7.4	29.6
**F22**	22.2	22.2
**F23**	0	29.6
**F24**	7.4	29.6
**F25**	22.2	14.8
**F26**	0	29.6
**F27**	29.6	7.4
**F28**	14.8	37
**F29**	/	/
**F30**	22.2	14.8
*^a^* Metalaxyl	59.3	66.7

*^a^* Metalaxyl at 10 mg/L; *^b^* “/” means not tested.

## 3. Experimental Section

### 3.1. General Synthesis

All anhydrous solvents were dried and purified by using standard techniques. The synthetic routes are given in Figure 2 and Scheme 1.

#### Synthetic Procedure for the Precursors of Target Compounds **F1**–**30**

Synthesis of *N*-ethyl-1-phenyl-9*H*-pyrido[3,4-*b*]indole-3-formamide (**F1**). l-Tryptamine (20.40 g, 0.1 mol) was added in three neck flasks, and 100 mL of acetic acid was added with stirring and dissolved by heating. Press material quality 1:1 ratio dropping weighed benzaldehyde (10.62 g, 0.1 mol), the reaction was heated to reflux, and the reaction progress was followed by thin layer chromatography (TLC). When TLC showed the point of material starting to disappear, we stopped the reaction. The resulting solution was cooled by suction filtration, and washed with water as eluent to remove acetic acid and give1-phenyl-1,2,3,4-tetrahydro-9*H*-pyrido[3,4-*b*]indole-3-carboxylic acid [21,26,27,28,29,30] as a white powdery solid (24.62 g, 85.27%).

Four milliliters of SOCl_2_ were slowly dropped into 40 mL of methanol in the ice salt bath, and then the ice bath was removed after the mixture was stirred for 20 min. Previous product 1-phenyl-1,2,3,4-tetrahydro-9*H*-pyrido[3,4-*b*]indole-3-carboxylic acid 11.68 g was added to the reaction mixture, then warmed slowly to reflux and the reaction was refluxed for 4 h, using TLC to track the progress of the reaction. When the reaction was completed, the reaction solution was stirred into 500 mL of ice water after it was cooled, and the pH was adjusted with 30% NaOH to neutral; at this time, the solution appeared as a heavy white precipitate. The precipitate was washed with water and filtered, then dried in a vacuum to give 1-phenyl-1,2,3,4-tetrahydro-9*H*-pyrido[3,4-*b*]indole-3-carboxylic acid methyl ester as a white powdery solid (9.26 g, 65.20%).

The product of the previous step was 1-phenyl-1,2,3,4-tetrahydro-9*H*-pyrido[3,4-*b*]indole-3-carboxylic acid methyl ester, of which 6.84 g was added in three neck flasks that were assembled in the ice salt bath; DMF was used as menstruum and KMnO_4_ was added according to the molar ratio of 1:1.2 ratio, reacting for 0.5 h in the ice bath. The reaction progress was followed by TLC. The mixture was cooled to room temperature after the reaction was finished, and then filtered in a vacuum after stirring for 12 h. The filtrate was added to water and stirred sufficiently to give the precipitated solid, the solution was filtered by suction, then the solid was washed with water to give1-phenyl-9*H*-pyrido[3,4-*b*]indole-3-carboxylic acid methyl ester as a pale yellow solid (3.66 g, 52.71%).

1-Phenyl-9*H*-pyrido[3,4-*b*]indole-3-carboxylic acid methyl ester (6.13 g) and ethanol (25.0 mL) were added into a 100 mL flask, then the flask was heated in an oil bath with stirring, and then 1 mL aqueous solution containing 0.5 g of sodium hydroxide was added dropwise in the pot. The, reaction was heated for 2 h and followed by TLC. Dilute hydrochloric acid was added dropwise until pH = 5–6 after completion of the reaction; the mixture was cooled in a refrigerator at 4 °C overnight, then filtered, washed with water, and dried to give *1*-phenyl-9*H*-pyrido[3,4-*b*]indole-3-carboxylic acid as a yellow solid (4.51 g, 65.1%).

1-phenyl-9*H*-pyrido[3,4-*b*]indole-3-carboxylic acid (5.76 g) was added to a 50 mL flask that was installed in a reflux condenser tube, drier, and exhaust absorption device, then 20 mL of thionyl chloride was added to the flask with stirring and the mixture was heated at reflux for 1 h. The condensed reflux into recovery device after the reaction was completed, and then the excess thionyl chloride was distilled off to give1-phenyl-9*H*-pyrido[3,4-*b*]indole-3-carbonyl chloride as an off-white solid powder (3.74 g, 67.1%).

The solid that was obtained at the previous step was dissolved with dichloromethane, then the solution, under an ice bath with stirring, was added dropwise slowly to a 50-mL single neck flask that had 1.5 g 30% ethylamine solution mixed with 2.0 g of alcohol of triethylamine. The mixture was stirred at room temperature for 30 min, and evaporated to dryness by rotary evaporation of dichloromethane, then dissolved in an appropriate amount of methanol. After that the solution was poured into water and cooled with stirring to sufficiently precipitate solid, and then the mixture was filtered. The resulting residue was washed with the amount of 5% sodium hydroxide solution and water until neutral, and finally dried to give **F1** as a yellow solid.

The target compounds **F2**–**30** were prepared by following the same procedure as for compound **F1**. The corresponding starting materials were all commercially available.

### 3.2. Spectral Data

*N-Ethyl-1-phenyl-9H-pyrido[3,4-b]indole-3-formamide* (C_20_H_17_N_3_O, m.w. 315) (**F1**): yield, 79.54%; m.p. 210–227 °C. IR (KBr) cm^−1^: 3430 (ν_N-H_), 2924, 2850 (ν_C-H_), 1647 (ν_C=O_), 1023 (ν_C-N_); ^1^H-NMR (600 MHz, DMSO) δ 11.82 (1H, s, N(9)H, c), 8.82 (1H, s, C(4)H, c), 8.70 (1H, t, *J* = 6.0 Hz, CO-NH), 8.43–8.39 (1H, m, C(5)H, c), 8.15 (2H, dd, *J* = 12.3, 5.2 Hz, Ph(2,6)H), 8.03–8.01 (1H, m, C(8)H, c), 7.65 (2H, dd, *J* = 7.4, 6.3 Hz, C(7)H, c, Ph(4)H), 7.60–7.57 (2H, m, Ph(3,5)H), 7.34–7.31 (1H, m, C(6)H, c), 3.44–3.40 (2H, m, CH_2_), 1.18 (3H, t, *J* = 7.2 Hz, CH_3_).

*N,N-Diethyl-1-phenyl-9H-pyrido[3,4-b]indole-3-formamide* (C_22_H_21_N_3_O, m.w. 343) (**F2**): yield, 57.1%; a pale yellow solid. m.p. 210–227 °C. IR (KBr) cm^−1^: 3449 (ν_N-H_), 2918, 2851 (ν_C-H_), 1623 (ν_C=O_), 1022 (ν_C-N_); ^1^H-NMR (600 MHz, DMSO) δ 11.71 (1H, s, N(9)H, c), 8.42 (1H, s, C(4)H, c), 8.35 (1H, d, *J* = 7.8 Hz, C(5)H, c), 8.02 (2H, d, *J* = 7.4 Hz, Ph(2,6)H), 7.67 (1H, d, *J* = 8.2 Hz, C(8)H, c), 7.63 (2H, t, *J* = 7.6 Hz, Ph(3,5)H), 7.56 (2H, m, *J* = 19.5, 7.5 Hz, C(7)H, c, Ph(4)H), 7.29 (1H, t, *J* = 7.4 Hz, C(6)H, c), 3.50 (4H, m, *J* = 13.7, 6.8 Hz, CH_2_), 1.23–1.18 (6H, m, CH_3_).

*N-Isopropyl-1-phenyl-9H-pyrido[3,4-b]indole-3-formamide* (C_21_H_19_N_3_O, m.w. 329) (**F3**): yield, 47.27%; a pale yellow solid. m.p. 188–191 °C. IR (KBr) cm^−1^: 3430 (ν_N-H_), 2924, 2850 (ν_C-H_), 1647 (ν_C=O_), 1252 (ν_C-N_); ^1^H-NMR (600 MHz, DMSO) δ 11.82 (1H, s, N(9)H, c), 8.82 (1H, s, C(4)H, c), 8.41 (1H, d, *J* = 7.9 Hz, CO-NH), 8.28 (1H, d, *J* = 8.3 Hz, C(5)H, c), 8.12 (2H, d, *J* = 7.3 Hz, Ph(2,6)H), 7.67 (3H, dd, *J* = 17.0, 8.1 Hz, C(8)H, c, Ph(3,5)H), 7.59 (2H, dd, *J* = 17.0, 7.7 Hz, C(7)H, c, Ph(4)H), 7.35–7.23 (1H, m, C(6)H, c), 4.20 (1H, m, *J* = 13.4, 6.7 Hz, CH), 1.26 (6H, d, *J* = 6.6 Hz, CH_3_).

*N-(2-Pyridyl)-1-phenyl-9H-pyrido[3,4-b]indole-3-formamide* (C_23_H_16_N_4_O, m.w. 364) (**F4**): yield, 81.49%; a yellow-brown solid. m.p. 182–183 °C. IR (KBr) cm^−1^: 3447, 3423 (ν_N-H_), 1623 (ν_C=O_), 1245 (ν_C-N_), 1493 (ν_C=C_); ^1^H-NMR (600 MHz, DMSO) δ 12.05 (1H, s, N(9)H, c), 10.66 (1H, s, CO-NH), 9.05 (1H, s, C(4)H, c), 8.88 (1H, s, C(5)H, c), 8.49 (1H, d, *J* = 7.6 Hz, Py(3)H), 8.39 (2H, d, *J* = 6.5 Hz, Ph(2,6)H), 8.35 (1H, d, *J* = 7.8 Hz, C(8)H, c), 8.13 (1H, d, *J* = 5.7 Hz, Py(5)H), 7.72 (1H, s, C(7)H, c), 7.60 (2H, d, *J* = 7.3 Hz, Ph(3,5)H), 7.35 (2H, d, *J* = 7.7 Hz, Ph(4)H, C(6)H, c), 7.31 (1H, d, *J* = 6.8 Hz, Py(4)H), 7.20 (1H, s, Py(6)H).

*N-(2-Chlorophenyl)-1-phenyl-9H-pyrido[3,4-b]indole-3-formamide* (C_24_H_16_ClN_3_O, m.w. 397) (**F5**): yield, 56.98%; a brown-yellow solid. m.p. 206–207 °C. IR (KBr) cm^−1^: 3424 (ν_N-H_), 1657 (ν_C=O_), 1497 (ν_C=C_), 742 (ν_C-Cl_); ^1^H-NMR (600 MHz, DMSO) δ 12.07 (1H, s, N(9)H, c), 11.06 (1H, s, CO-NH), 9.02 (1H, s, C(4)H, c), 8.63–8.60 (1H, m, C(5)H, c), 8.49 (1H, d, *J* = 7.8 Hz, Ph_2_(6)H), 8.19 (2H, d, *J* = 7.2 Hz, Ph_1_(2,6)H), 7.73 (1H, d, *J* = 8.2 Hz, C(8)H, c), 7.70 (2H, t, *J* = 7.6 Hz, Ph_1_(3,5)H), 7.67–7.61 (2H, m, Ph_2_(3)H, C(7)H, c), 7.61–7.60 (1H, m, Ph_1_(4)H), 7.45 (1H, t, *J* = 7.7 Hz, Ph_2_(4)H), 7.36 (1H, t, *J* = 7.4 Hz, C(6)H, c), 7.20–7.17 (1H, m, Ph_2_(4)H).

*N-Ethyl-1-(4-nitrophenyl)-9H-pyrido[3,4-b]indole-3-formamide* (C_20_H_16_N_4_O_3_, m.w. 360) (**F6**): yield, 77.82%; a bright yellow solid. m.p. 269–272 °C. IR (KBr) cm^−1^: 3435 (ν_N-H_), 2921, 2848 (ν_C-H_), 1624 (ν_C=O_), 1249 (ν_C-N_), 1520, 1347 (ν_NO2_); ^1^H-NMR (600 MHz, DMSO) δ 12.20 (1H, s, N(9)H, c), 9.02 (1H, s, C(4)H, c), 8.48 (2H, d, *J* = 3.1 Hz, Ph(2,6)H), 8.47–8.46 (2H, m, Ph(3,5)H), 8.46–8.44 (1H, m, C(5)H, c), 8.32 (1H, d, *J* = 8.7 Hz, CO-NH), 7.72 (1H, t, *J* = 7.8 Hz, C(8)H, c), 7.63 (1H, dd, *J* = 11.9, 4.6 Hz, C(7)H, c), 7.37–7.34 (1H, m, C(6)H, c), 2.80 (2H, m, *J* = 11.9, 6.0 Hz, CH_2_), 1.14 (3H, t, *J* = 7.3 Hz, CH_3_).

*N,N-Diethyl-1-(4-nitrophenyl)-9H-pyrido[3,4-b]indole-3-formamide* (C_22_H_20_N_4_O_3_, m.w. 388) (**F7**): yield, 66.08%; a yellow-brown solid. m.p. 252–254 °C. IR (KBr) cm^−1^: 3446 (ν_N-H_), 2925, 2851 (ν_C-H_), 1608 (ν_C=O_), 1105 (ν_C-N_), 1515, 1342 (ν_NO2_); ^1^H-NMR (600 MHz, DMSO) δ 11.97 (1H, s, N(9)H, c), 8.53 (1H, s, C(4), c), 8.46 (2H, d, *J* = 8.8 Hz, Ph(2,6)H), 8.39 (1H, d, *J* = 7.9 Hz, C(5)H, c), 8.31 (2H, d, *J* = 8.6 Hz, Ph(3,5)H), 7.69 (1H, d, *J* = 8.1 Hz, C(8)H, c), 7.61 (1H, t, *J* = 7.7 Hz, C(7)H, c), 7.32 (1H, t, *J* = 7.5 Hz, C(6)H, c), 3.53–3.46 (4H, m, CH_2_), 1.24–1.19 (6H, m, CH_3_).

*N-Isopropyl-1-(4-nitrophenyl)-9H-pyrido[3,4-b]indole-3-formamide* (C_21_H_18_N_4_O_3_, m.w. 374) (**F8**): yield, 84.58%; a pale yellow solid. m.p. 260–262 °C. IR (KBr) cm^−1^: 3426 (ν_N-H_), 2918, 2850 (ν_C-H_), 1652 (ν_C=O_), 1249 (ν_C-N_), 1519, 1345 (ν_NO2_); ^1^H-NMR (600 MHz, DMSO) δ 12.02 (1H, s, N(9)H, c), 8.91 (1H, s, C(4)H, c), 8.47 (2H, d, *J* = 4.8 Hz, Ph(2,6)H), 8.43 (2H, d, *J* = 8.7 Hz, Ph(3,5)H), 8.34 (1H, d, *J* = 8.4 Hz, C(5)H, c), 8.31 (1H, d, *J* = 8.6 Hz, CO-NH), 7.69 (1H, d, *J* = 8.9 Hz, C(8)H, c), 7.66–7.63 (1H, m, C(7)H, c), 7.35 (1H, d, *J* = 7.2 Hz, C(6)H, c), 4.22 (1H, m, *J* = 13.5, 6.7 Hz, CH), 1.26 (6H, d, *J* = 6.6 Hz, CH_3_).

*N-(2-Pyrido)-1-(4-nitrophenyl)-9H-pyrido[3,4-b]indole-3-formamide* (C_23_H_15_N_5_O_3_, m.w. 409) (**F9**): yield, 62.69%; a yellow-brown solid. m.p. 253–255 °C. IR (KBr) cm^−1^: 3427 (ν_N-H_), 1625 (ν_C=O_), 1321 (ν_C-N_), 1601, 1495 (ν_C=C_), 1519, 1348 (ν_NO2_); ^1^H-NMR (600 MHz, DMSO) δ 12.15 (1H, s, N(9)H, c), 10.61 (1H, s, CO-NH), 9.02 (1H, s, C(4)H, c), 8.53 (1H, d, *J* = 7.0 Hz, C(5)H, c), 8.47 (2H, d, *J* = 4.2 Hz, Ph(2,6)H), 8.31 (2H, d, *J* = 8.7 Hz, Ph(3,5)H), 8.17 (1H, d, *J* = 8.7 Hz, Py(3)H), 7.84 (1H, d, *J* = 8.7 Hz, C(5)H, c), 7.72 (1H, d, *J* = 2.7 Hz, Py(5)H), 7.68–7.65 (1H, m, C(7)H, c), 7.61–7.57 (1H, m, C(6)H, c), 7.37 (1H,d, *J* = 7.5 Hz, Py(4)H), 7.35–7.29 (1H, m, Py(6)H).

*N-(2-Chlorophenyl)-1-(4-nitrophenyl)-9H-pyrido[3,4-b]indole-3-formamide* (C_24_H_15_ClN_4_O_3_, m.w. 442) (**F10**): yield, 43.6%; a yellow-brown solid. m.p. 277–278 °C. IR (KBr) cm^−1^: 3439 (ν_N-H_), 1629 (ν_C=O_), 1495 (ν_C=C_), 737 (ν_C-Cl_), 1519, 1348 (ν_NO2_); ^1^H-NMR (600 MHz, DMSO) δ 12.15 (1H, s, N(9)H, c), 10.61 (1H, s, CO-NH), 9.02 (1H, s, C(4)H, c), 8.53 (1H, d, *J* = 6.8 Hz, C(5)H, c), 8.47 (2H, d, *J* = 4.2 Hz, Ph_1_(2,6)H), 8.43 (1H, d, *J* = 8.7 Hz, Ph_2_(6)H), 8.39–8.37 (1H, m, Ph_2_(3)H), 8.31 (2H, d, *J* = 8.7 Hz, Ph_1_(3,5)H), 8.17 (1H, d, *J* = 8.7 Hz, C(8)H, c), 7.84 (1H, d, *J* = 8.7 Hz, C(7)H, c), 7.72 (1H, d, *J* = 2.7 Hz, Ph_2_(5)H), 7.68–7.65 (1H, m, C(6)H, c), 7.37 (1H, d, *J* = 7.5 Hz, Ph_2_(4)H).

*N-Ethyl-1-(4-methoxyphenyl)-9H-pyrido[3,4-b]indole-3-formamide* (C_21_H_19_N_3_O_2_, m.w. 345) (**F11**): yield, 41.48%; a pale yellow solid. m.p. 171–173 °C. IR (KBr) cm^−1^: 3429 (ν_N-H_), 2923, 2853 (ν_C-H_), 1653 (ν_C=O_), 1249 (ν_C-N_), 1249, 1119 (ν_C-O-C_); ^1^H-NMR (600 MHz, DMSO) δ 11.80 (1H, s, N(9)H, c), 8.76 (1H, s, C(4)H, c), 8.69 (1H, t, *J* = 6.1 Hz, CO-NH), 8.39 (1H, t, *J* = 7.2 Hz, C(5)H, c), 8.13 (2H, dd, *J* = 9.2, 2.4 Hz, Ph(2,6)H), 7.69 (1H, d, *J* = 8.2 Hz, C(8)H, c), 7.58 (1H, dd, *J* = 11.7, 4.6 Hz, C(7)H, c), 7.31 (1H, dd, *J* = 14.3, 7.1 Hz, C(6)H, c), 7.19 (2H, dd, *J* = 9.8, 6.3 Hz, Ph(3,5)H), 3.89 (3H, s, *J* = 2.9 Hz, OCH_3_), 3.45–3.38 (2H, m, CH_2_), 1.23–1.14 (3H, m, CH_3_).

*N,N-Diethyl-1-(4-methoxyphenyl)-9H-pyrido[3,4-b]indole-3-formamide* (C_23_H_23_N_3_O_2_, m.w. 373) (**F12**): yield, 36.66%; a pale yellow solid. m.p. 225–226 °C. IR (KBr) cm^−1^: 3427(ν_N-H_), 2921, 2851 (ν_C-H_), 1609 (ν_C=O_), 1243 (ν_C-N_), 1243, 1113 (ν_C-O-C_); ^1^H-NMR (600 MHz, DMSO) δ 11.67 (1H, s, N(9)H, c), 8.36 (1H, s, C(4)H, c), 8.35–8.32 (1H, m, C(5)H, c), 7.99 (2H, d, *J* = 8.7 Hz, Ph(2,6)), 7.67 (1H, d, *J* = 8.2 Hz, C(8)H, c), 7.57 (1H, t, *J* = 7.6 Hz, C(7)H, c), 7.28 (1H, t, *J* = 7.5 Hz, C(6)H, c), 7.19 (2H, d, *J* = 8.7 Hz, Ph(3,5)H), 3.88 (s, 3H, OCH_3_), 3.49 (4H, m, *J* = 13.6, 6.8 Hz, CH_2_), 1.23–1.18 (6H, m, CH_3_).

*N-Isopropyl-1-(4-methoxyphenyl)-9H-pyrido[3,4-b]indole-3-formamide* (C_22_H_21_N_3_O_2_, m.w. 359) (**F13**): yield, 23.92%; a yellow solid. m.p. 188–189 °C. IR (KBr) cm^−1^: 3430 (ν_N-H_), 2924, 2850 (ν_C-H_), 1648 (ν_C=O_), 1176 (ν_C-N_), 1248, 1176 (ν_C-O-C_); ^1^H-NMR (600 MHz, DMSO) δ 11.78 (1H, s, N(9)H, c), 8.77 (1H, s, C(4)H, c), 8.39 (1H, d, *J* = 7.9 Hz, C(5)H, c), 8.28 (1H, d, *J* = 8.3 Hz, CO-NH), 8.09 (2H, dd, *J* = 6.8, 4.8 Hz, Ph(2,6)H), 7.68 (1H, dd, *J* = 8.6, 2.1 Hz, C(8)H, c), 7.60−7.58 (1H, m, C(7)H, c), 7.37−7.26 (1H, m, C(6)H, c), 7.22−7.20 (2H, m, Ph(3,5)H), 4.21−4.18 (1H, m, CH), 3.89 (3H, s, *J* = 0.9 Hz, OCH_3_), 1.26 (6H, dd, *J* = 6.6, 1.4 Hz, CH_3_).

*N-(2-Pyridyl)-1-(4-methoxyphenyl)-9H-pyrido[3,4-b]indole-3-formamide* (C_24_H_18_N_4_O_2_, m.w. 394) (**F14**): yield, 40.36%; a brown solid. m.p. 244–245 °C. IR (KBr) cm^−1^: 3414, 3340 (ν_N-H_), 1686 (ν_C=O_), 1300 (ν_C-N_), 1608, 1512 (ν_C=C_), 1252, 1176 (ν_C-O-C_); ^1^H-NMR (600 MHz, DMSO) δ 12.14 (1H, s, N(9)H, c), 10.66 (1H, s, CO-NH), 9.05 (1H, s, C(4)H, c), 8.40 (2H, d, *J* = 4.9 Hz, Py(3)H, C(5)H, c), 8.09 (2H, t, *J* = 8.6 Hz, Ph(2,6)H), 7.94–7.91 (2H, m, Py(5)H, C(8), c), 7.73–7.71 (1H, m, C(7)H, c), 7.64–7.62 (1H, m, C(6)H, c), 7.28–7.25 (2H, m, Ph(3,5)H), 6.98 (1H, d, *J* = 8.8 Hz, Py(4)H), 6.88–6.83 (1H, m, Py(6)H), 3.91 (3H, t, *J* = 2.8 Hz, OCH_3_).

*N-(2-Chlorophenyl)-1-(4-methoxyphenyl)-9H-pyrido[3,4-b]indole-3-formamide* (C_25_H_18_ClN_3_O_2_, m.w. 427) (**F15**): yield, 7.66%; a yellow-brown solid. m.p. 250–251 °C. IR (KBr) cm^−1^: 3422 (ν_N-H_), 1665 (ν_C=O_), 1510 (ν_C=C_), 746 (ν_C-Cl_), 1249, 1114 (ν_C-O-C_); ^1^H-NMR (600 MHz, DMSO) δ 12.02 (1H, s, N(9)H, c), 11.08 (1H, s, CO-NH), 8.98 (1H, d, *J* = 13.0 Hz, C(4)H, c), 8.62 (1H, d, *J* = 8.1 Hz, C(5)H, c), 8.47 (1H, d, *J* = 7.7 Hz, Ph_2_(6)H), 8.15 (2H, d, *J* = 8.8 Hz, Ph_1_(2,6)H), 7.74–7.71 (1H, m, C(7)H, c), 7.62 (2H, dd, *J* = 16.7, 8.0 Hz, C(8)H, c, Ph_2_(3)H), 7.46 (1H, t, *J* = 7.8 Hz, C(6)H, c), 7.35 (1H, t, *J* = 7.5 Hz, Ph_2_(5)H), 7.25 (2H, d, *J* = 8.6 Hz, Ph_1_(3,5)H), 7.19 (1H, t, *J* = 7.7 Hz, Ph_2_(4)H), 3.91 (3H, s, OCH_3_).

*N-Ethyl-1-(3,4,5-trimethoxyphenyl)-9H-pyrido[3,4-b]indole-3-formamide* (C_23_H_23_N_3_O_4_, m.w. 405) (**F16**): yield, 54.13%; a pale yellow solid. m.p. 241–243 °C. IR (KBr) cm^−1^: 3429 (ν_N-H_), 2925, 2850 (ν_C-H_), 1653 (ν_C=O_), 1250 (ν_C-N_), 1250, 1104 (ν_C-O-C_); ^1^H-NMR (600 MHz, DMSO) δ 11.73 (1H, s, N(9)H, c), 8.92 (1H, d, *J* = 20.2 Hz, C(4)H, c), 8.41 (1H, t, *J* = 7.9 Hz, CO-NH), 7.58 (3H, dd, *J* = 11.5, 8.2 Hz, Ph(2,6)H, C(8)H, c), 7.30 (2H, ddd, *J* = 20.1, 13.5, 7.1 Hz, C(7)H, c, C(6)H, c), 4.02 (3H, s, Ph_1_(4)OCH_3_), 3.93 (6H, s, Ph_1_(3,5)OCH_3_), 3.17 (2H,d, *J* = 5.2 Hz, CH_2_), 1.15 (3H,d, *J* = 2.0 Hz, CH_3_).

*N,N-Diethyl-1-(3,4,5-trimethoxyphenyl)-9H-pyrido[3,4-b]indole-3-formamide* (C_25_H_27_N_3_O_4_, m.w. 433) (**F17**): yield, 14.84%; a pale yellow solid. m.p. 268–270 °C. IR (KBr) cm^−1^: 3454 (ν_N-H_), 2929, 2850 (ν_C-H_), 1623 (ν_C=O_), 1238 (ν_C-N_), 1238, 1127 (ν_C-O-C_); ^1^H-NMR (600 MHz, DMSO) δ 11.72 (1H, s, N(9)H, c), 8.90 (1H, s, C(4)H, c), 8.42 (1H, d, *J* = 7.8 Hz, C(5)H, c), 8.13 (1H, d, *J* = 8.4 Hz, C(8)H, c), 7.64–7.54 (3H, m, C(7)H, c, Ph(2,6)H), 7.31 (1H, dd, *J* = 11.4, 4.4 Hz, C(6)H, c), 4.03 (3H, s, Ph_1_(4)OCH_3_), 3.93 (6H, s, Ph_1_(3,5)OCH_3_), 3.31–3.03 (4H, m, CH_2_), 1.21 (6H, t, *J* = 6.5 Hz, CH_3_).

*N-Isopropyl-1-(3,4,5-trimethoxyphenyl)-9H-pyrido[3,4-b]indole-3-formamide* (C_24_H_25_N_3_O_4_, m.w. 419) (**F18**): yield, 22.55%; a yellow solid. m.p. 311–317 °C. IR (KBr) cm^−1^: 3433 (ν_N-H_), 2925, 2855 (ν_C-H_), 1651 (ν_C=O_), 1052 (ν_C-N_), 1252, 1116 (ν_C-O-C_); ^1^H-NMR (600 MHz, DMSO) δ 11.72 (1H, s, N(9)H, c), 9.03–8.72 (1H, m, CO-NH), 8.44–8.41 (1H, m, C(4)H, c), 8.35 (1H, d, *J* = 7.9 Hz, C(5)H, c), 7.67 (1H, dd, *J* = 17.8, 8.2 Hz, C(8), c), 7.61–7.56 (1H, m, C(7)H, c), 7.29–7.27 (1H, m, C(6)H, c), 7.23 (2H, s, Ph(2,6)H), 3.93–3.90 (6H, m, Ph_1_(3,5)OCH_3_), 3.78 (3H, d, *J* = 3.0 Hz, Ph_1_(4)OCH_3_), 3.50 (1H, s, CH), 1.23 (6H, m, *J* = 12.5, 7.0 Hz, CH_3_).

*N-(2-Pyridyl)-1-(3,4,5-trimethoxyphenyl)-9H-pyrido[3,4-b]indole-3-formamide* (C_26_H_22_N_4_O_4_, m.w. 454) (**F19**): yield, 41.63%; a khaki solid. m.p. 279–281 °C. IR (KBr) cm^−1^: 3433 (ν_N-H_), 1651 (ν_C=O_), 1252 (ν_C-N_), 1623, 1525 (ν_C=C_), 1252, 1113 (ν_C-O-C_); ^1^H-NMR (600 MHz, DMSO) δ 11.89 (1H, s, N(9)H, c), 10.58 (1H, s, CO-NH), 9.09 (1H, s, C(4)H, c), 8.50–8.48 (1H, m, C(5)H, c), 8.37 (1H, d, *J* = 8.3 Hz, Py(3)H), .94 (2H, dd, *J* = 16.5, 8.4 Hz, C(8)H, c, Py(5)H), 7.63 (2H, d, *J* = 3.1 Hz, C(7)H, c, C(6), c), 7.35 (2H, ddd, *J* = 9.6, 6.2, 3.9 Hz, Py(4,6)H), 7.22–7.19 (2H, m, Ph(2,6)H), 3.95–3.92 (6H, m, Ph(3,5)OCH_3_), 3.87 (3H, s, Ph(4)OCH_3_).

*N-(2-Chlorophenyl)-1-(3,4,5-trimethoxyphenyl)-9H-pyrido[3,4-b]indole-3-formamide* (C_27_H_22_ClN_3_O_4_, m.w. 487) (**F20**): yield, 10.09%; a bright yellow solid. m.p. 275–278 °C. IR (KBr) cm^−1^: 3429 (ν_N-H_), 1624 (ν_C=O_), 1484 (ν_C=C_), 749 (ν_C-Cl_), 1250, 1109 (ν_C-O-C_); ^1^H-NMR (600 MHz, DMSO) δ 12.11 (1H, s, N(9)H, c), 11.07 (1H, s, CO-NH), 9.54–9.53 (1H, m, C(4)H, c), 9.06 (1H, s, Ph_2_(6)H), 9.01 (1H, s, C(5)H, c), 8.51–8.47 (2H, m, C(8), c, Ph_2_(6)H), 7.64 (2H, s, C(7), c, C(6)H, c), 7.41 (2H, s, Ph_1_(2,6)H), 7.26 (1H, s, Ph_2_(5)H), 7.19–7.18 (1H, m, Ph_2_(4)H), 3.97–3.92 (6H, m, Ph_1_(3,5)OCH_3_), 3.80 (3H, s, Ph_1_(4)OCH_3_).

*N-Ethyl-1-(4-trifluoromethylphenyl)-9H-pyrido[3,4-b]indole-3-formamide* (C_21_H_16_F_3_N_3_O, m.w. 383) (**F21**): yield,78.1%; a beige solid. m.p. 261–263 °C. IR (KBr) cm^−1^: 3428 (ν_N-H_), 2926, 2848 (ν_C-H_), 1625 (ν_C=O_), 1250 (ν_C-N_), 1119 (ν_CF3_); ^1^H-NMR (600 MHz, DMSO) δ 11.95 (1H, s, N(9)H, c), 8.87 (1H, s, CO-NH), 8.76 (1H, t, *J* = 6.1 Hz, C(4)H, c), 8.40 (2H, dd, *J* = 35.2, 8.0 Hz, Ph(2,6)H), 7.99 (2H, d, *J* = 8.2 Hz, Ph(3,5)H), 7.68 (1H, d, *J* = 8.2 Hz, C(8)H, c), 7.65–7.58 (1H, m, C(7)H, c), 7.43–7.27 (1H, m, C(6)H, c), 3.38 (2H, q, *J* = 4.3 Hz, CH_2_), 1.18 (3H, t, *J* = 7.2 Hz, CH_3_).

*N,N-Diethyl-1-(4-trifluoromethylphenyl)-9H-pyrido[3,4-b]indole-3-formamide* (C_23_H_20_F_3_N_3_O, m.w. 411) (**F22**): yield, 83.4%; a beige solid. m.p. 238–239 °C. IR (KBr) cm^−1^: 3435 (ν_N-H_), 2927, 2851 (ν_C-H_), 1615 (ν_C=O_), 1323 (ν_C-N_), 1122 (ν_CF3_); ^1^H-NMR (600 MHz, DMSO) δ 11.83 (1H, s, N(9)H, c), 8.49 (1H, s, C(4)H, c), 8.38 (1H, d, *J* = 7.9 Hz, C(5)H, c), 8.22 (2H, d, *J* = 8.1 Hz, Ph(2,6)H), 7.99 (2H, d, *J* = 8.2 Hz, Ph(3,5)H), 7.66 (1H, d, *J* = 8.2 Hz, C(8)H, c), 7.60 (1H, t, *J* = 7.6 Hz, C(7)H, c), 7.31 (1H, t, *J* = 7.4 Hz, C(6)H, c), 3.53–3.47 (4H, m, CH_2_), 1.20 (6H, t, *J* = 14.3, 7.0 Hz, CH_3_).

*N-Isopropyl-1-(4-trifluoromethylphenyl)-9H-pyrido[3,4-b]indole-3-formamide* (C_22_H_18_F_3_N_3_O, m.w. 397) (**F23**): yield, 69.3%; a beige solid. m.p. 245–247 °C. IR (KBr) cm^−1^: 3437 (ν_N-H_), 2925, 2854 (ν_C-H_), 1646 (ν_C=O_), 1325 (ν_C-N_), 1122 (ν_CF3_); ^1^H-NMR (600 MHz, DMSO) δ 11.95 (1H, s, N(9)H, c), 8.88 (1H, s, C(4)H, c), 8.43 (1H, d, *J* = 7.9 Hz, C(5)H, c), 8.33 (3H, d, *J* = 8.0 Hz, Ph(3,5), C(8)H, c), 8.00 (2H, d, *J* = 8.2 Hz, Ph(3,5)H), 7.68 (1H, d, *J* = 8.2 Hz, CO-NH), 7.64–7.58 (1H, m, C(7)H, c), 7.33 (1H, dd, *J* = 11.3, 4.2 Hz, C(6)H, c), 4.21 (1H, qd, *J* = 13.3, 6.6 Hz, CH), 1.25 (6H, d, *J* = 6.6 Hz, CH_3_).

*N-(2-Pyridyl)-1-(4-trifluoromethylphenyl)-9H-pyrido[3,4-b]indole-3-formamide* (C_24_H_15_F_3_N_4_O, m.w. 432) (**F24**): yield, 57.7%; a beige solid. m.p. 269–271 °C. IR (KBr) cm^−1^: 3438 (ν_N-H_), 1627 (ν_C=O_), 1322 (ν_C-N_), 1524 (ν_C=C_), 1121 (ν_CF3_); ^1^H-NMR (600 MHz, DMSO) δ 12.15 (1H, s, N(9)H, c), 10.61 (1H, d, *J* = 13.9 Hz, CO-NH), 9.10 (1H, s, C(4)H, c), 8.51 (1H, d, *J* = 7.9 Hz, C(5)H, c), 8.38 (2H, ddd, *J* = 12.1, 6.9, 3.6 Hz, Py(3), C(8)H, c), 8.34 (2H, d, *J* = 8.3 Hz, Ph(2,6)H), 8.06 (2H, d, *J* = 8.1 Hz, Ph(3,5)H), 7.70 (2H, dd, *J* = 16.2, 8.2 Hz, C(7, 8)H, c), 7.62–7.57 (1H, m, C(6)H, c), 7.41–7.35 (1H, m, Py(4)H), 7.26–7.14 (1H, m, Py(6)H).

*N-(2-Chlorophenyl)-1-(4-trifluoromethylphenyl)-9H-pyrido[3,4-b]indole-3-formam-ide* (C_25_H_15_ClF_3_N_3_O, m.w. 465) (**F25**): yield, 95.6%; a beige solid. m.p. 295–297 °C.IR (KBr) cm^−1^: 3442 (ν_N-H_), 1641 (ν_C=O_), 1539 (ν_C=C_), 744 (ν_C-Cl_), 1116 (ν_CF3_); ^1^H-NMR (600 MHz, DMSO) δ 12.17 (1H, s, N(9)H, c), 10.96 (1H, s, CO-NH), 9.05 (1H, s, C(4)H, c), 8.56 (1H, dd, *J* = 8.2, 1.5 Hz, C(5)H, c), 8.49 (1H, d, *J* = 7.9 Hz, Ph_2_(6)H), 8.37 (2H, d, *J* = 8.1 Hz, Ph_1_(2,6)H), 8.04 (2H, d, *J* = 8.2 Hz, Ph_1_(3,5)H), 7.72 (1H, d, *J* = 8.2 Hz, C(8)H, c), 7.67–7.63 (1H, m, Ph_2_(3)H), 7.59 (1H, dd, *J* = 8.0, 1.4 Hz, C(7)H, c), 7.50–7.42 (1H, m, Ph_2_(5)H), 7.41–7.33 (1H, m, C(6)H, c), 7.18 (1H, td, *J* = 7.8, 1.5 Hz, Ph_2_(4)H).

*N-Ethyl-1-(4-chlorophenyl)-9H-pyrido[3,4-b]indole-3-formamide* (C_20_H_16_ClN_3_O, m.w. 349) (**F26**): yield, 49.84%; a gray-yellow solid. m.p. 234–236 °C. IR (KBr) cm^−1^: 3440 (ν_N-H_), 2926, 2855 (ν_C-H_), 1644 (ν_C=O_), 1091 (ν_C-N_), 746 (ν_C-Cl_); ^1^H-NMR (600 MHz, DMSO) δ 12.06 (1H, s, N(9)H, c), 8.93 (2H, d, *J* = 10.2 Hz, Ph_1_(2,6)H), 8.43 (1H, d, *J* = 7.8 Hz, CO-NH), 8.07–8.05 (2H, m, C(4, 5)H, c), 7.71–7.69 (3H, m, C(8)H, c, Ph_1_(3,5)H), 7.63–7.60 (1H, m, C(7)H, c), 7.34 (1H, t, *J* = 7.1 Hz, C(6)H, c), 3.93 (2H, m, CH_2_), 1.36–1.05 (3H, m, CH_3_).

*N,N-Diethyl-1-(4-chlorophenyl)-9H-pyrido[3,4-b]indole-3-formamide* (C_22_H_20_ClN_3_O, m.w. 377) (**F27**): yield, 25.64%; a pale yellow solid. m.p. 247–248 °C. IR (KBr) cm^−1^: 3436 (ν_N-H_), 2925, 2855 (ν_C-H_), 1626 (ν_C=O_), 1093 (ν_C-N_), 747 (ν_C-Cl_); ^1^H-NMR (600 MHz, DMSO) δ 11.88 (1H, s, N(9)H, c), 8.82 (1H, s, C(4)H, c), 8.41 (1H, d, *J* = 7.9 Hz, C(5)H, c), 8.21–8.19 (2H, m, Ph_1_(2,6)H), 7.70–7.68 (3H, m, C(8)H, c, Ph_1_(3,5)H), 7.62–7.59 (1H, m, C(7)H, c), 7.32 (1H, t, *J* = 7.1 Hz, C(6)H, c), 3.16 (4H, q, *J* = 5.2 Hz, CH_2_), 1.16 (6H, m, *J* = 26.0, 7.2 Hz, CH_3_).

*N-Isopropyl-1-(4-chlorophenyl)-9H-pyrido[3,4-b]indole-3-formamide* (C_21_H_18_ClN_3_O, m.w. 363) (**F28**): yield, 60.15%; a pale yellow solid. m.p. 257–259 °C. IR (KBr) cm^−1^: 3439 (ν_N-H_), 2969, 2927 (ν_C-H_), 1648 (ν_C=O_), 1092 (ν_C-N_), 746 (ν_C-Cl_); ^1^H-NMR (600 MHz, DMSO) δ 11.87 (1H, s, N(9)H, c), 8.85 (1H, d, *J* = 17.0 Hz, C(5)H, c), 8.45–8.28 (2H, m, Ph_1_(2,6)H), 8.22–8.07 (2H, m, C(4)H, c, CO-NH), 7.70 (3H, ddd, *J* = 15.4, 8.7, 5.3 Hz, C(8)H, c, Ph_1_(3,5)H), 7.64–7.56 (1H, m, C(8 )H, c), 7.37–7.28 (1H, m, C(6)H, c), 4.24–4.13 (1H, CH, m), 1.25 (6H, d, *J* = 6.6 Hz, CH_3_).

*N-(2-Pyridyl)-1-(4-chlorophenyl)-9H-pyrido[3,4-b]indole-3-formamide* (C_23_H_15_ClN_4_O, m.w. 398) (**F29**): yield, 7.29%; a pale yellow solid. m.p. 263–265 °C. IR (KBr) cm^−1^: 3436 (ν_N-H_), 1628 (ν_C=O_), 1455 (ν_C=C_), 1094 (ν_C-N_), 747 (ν_C-Cl_); ^1^H-NMR (600 MHz, DMSO) δ 12.03 (1H, s, N(9)H, c), 8.93 (1H, s, N(9)H, c), 8.43 (2H, d, *J* = 7.9 Hz, Ph_1_(2,6)H), 8.10 (3H, t, *J* = 8.5 Hz, C(4,5)H, c, Py(3)H), 7.70 (5H, t, *J* = 8.5 Hz, C(7,8)H, c, Ph_1_(3,5)H, Ph_2_(5)H), 7.62 (1H, t, *J* = 7.1 Hz, C(6)H, c), 7.32 (2H, dd, *J* = 28.9, 21.6 Hz, Ph_2_(4,6)H).

*N-(2-Chlorophenyl)-1-(4-chlorophenyl)-9H-pyrido[3,4-b]indole-3-formamide* (C_24_H_15_Cl_2_N_3_O, m.w. 432) (**F30**): yield, 35.84%; a pale yellow solid. m.p. 271–273 °C. IR (KBr) cm^−1^: 3449 (ν_N-H_), 1649 (ν_C=O_), 1594, 1497 (ν_C=C_), 1094 (ν_C-N_), 741 (ν_C-Cl_); ^1^H-NMR (600 MHz, DMSO) δ 12.16 (1H, s, N(9)H, c), 11.00 (1H, s, CO-NH), 9.02 (1H s, C(4)H, c), 8.58 (1H,t, *J* = 8.2, 4.1 Hz, C(5)H, c), 8.49 (1H, d, *J* = 7.9 Hz, Ph_2_(6)H), 8.24–8.17 (2H, m, Ph_1_(3,5)H), 7.78–7.72 (3H, m, Ph_1_(3,5)H, Ph_2_(3)H), 7.64 (1H, ddd, *J* = 8.2, 5.8, 1.1 Hz, C(8)H, c), 7.60 (1H, dd, *J* = 8.0, 1.4 Hz, C(7)H, c), 7.47–7.43 (1H, m, Ph_2_(5)H), 7.40–7.33 (1H, m, C(6)H, c), 7.22–7.16 (1H, m, Ph_2_(4)H).

### 3.3. Fungicidal Activities

The compounds were screened on a broader range of fungal pathogens in mycelial growth tests in artificial media against *Fusarium oxysporum* f.sp.cubense*,* *Colletotrichum gloeosporioides* (Penz.), *R. solani*, *Peronophthora litchii*, *Phytophthora nicotianae*, and *O. citriaurantii* ex Persoon, at the concentration of 100 μg/mL. Each assay contained three replicates for each concentration. The plates were stored in controlled environment cabinets for between two and seven days, depending on the assay, after which mycelia growth was assessed. Treatments were scored as percentage inhibition of mycelial growth relative to untreated controls. The biological data presented in Table 1 are the mean scores for each treatment across replicates.

### 3.4. Leaf-Piece Assays

The compounds were also evaluated in leaf-piece assays, at the concentration of 100 μg/mL for *P. litchi* on young leaves of litchi. The formulated chemicals were applied to leaf pieces prior to inoculation with spores of the pathogen, and also after they had been inoculated with pathogen spores. Each assay contained three replicates for each concentration. Assessments were made three days after inoculation, depending on the assay, after which disease inhibition was assessed. Treatments were scored as percentage inhibition of disease development relative to untreated controls. The biological data presented in Table 2 are the mean scores for each treatment across replicates.

## 4. Conclusions

In summary, a series of β-carbolines derivatives were designed, synthesized, and first assayed for their fungicidal activities *in vitro* and *in vivo*. The results showed that most of these compounds exhibited good fungicidal activities, and these compounds had certain selectivity toward fungi species. Of all the new compounds synthesized, **F4** (*N*-(2-pyridyl)-1-phenyl-9*H*-pyrido[3,4-*b*]indole-3-formamide) showed activity almost at the same level as the control compound metalaxyl against *P. litchii* and *P. nicotianae in vitro*. Very interestingly, this compound exhibited better activity than the control compound validamycin against *R. solani in vitro*, and also had excellent preventative activity against *P. litchi in vivo*. Further studies on the activity mechanism of these compounds are underway in our laboratory.
